# Unravelling the Genetic Architecture of Flowering in Common Vetch (*Vicia sativa* L.) Through Multi-Season QTL Mapping

**DOI:** 10.3390/biology15141209

**Published:** 2026-07-22

**Authors:** Eva M. Córdoba, Clara I. González-Verdejo, Sergio G. Atienza, Salvador Nadal, Carmen M. Ávila

**Affiliations:** 1 Área Mejora Vegetal y Biotecnología, Instituto Andaluz de Investigación y Formación Agraria, Pesquera, Alimentaria y de la Producción Ecológica (IFAPA)-Centro Alameda del Obispo, Avenida Menéndez Pidal s/n, 14004 Cordoba, Spain; evam.cordoba@juntadeandalucia.es (E.M.C.); clarai.gonzalez@juntadeandalucia.es (C.I.G.-V.); salvador.nadal@juntadeandalucia.es (S.N.); 2 Instituto de Agricultura Sostenible-CSIC, Avenida Menéndez Pidal s/n, 14004 Cordoba, Spain; sgatienza@ias.csic.es

**Keywords:** phenology, thermal time, DArTSeq, flowering duration, Mediterranean adaptation, genetic architecture, marker-assisted breeding, climate resilience

## Abstract

Flowering time is a key adaptive trait in common vetch (*Vicia sativa* L.), influencing crop adaptation, yield potential, and forage quality. Despite its importance, the genetic basis of flowering in this species remains poorly understood. In this study, we analyzed the genetic architecture of flowering-related traits in an F_2:3_ population under field conditions over two consecutive growing seasons. Four traits were evaluated using thermal time expressed as growing degree days (GDDs), and quantitative trait loci (QTL) analysis was conducted using a high-density genetic map derived from DArTSeq markers. The linkage map comprised 419 unique positions across six linkage groups, spanning 1728.5 cM. Ten QTLs associated with flowering traits were identified on chromosomes 2, 3, 4, and 6. Five QTLs were consistent across seasons, defining three major genomic regions controlling flowering. Additional season-specific QTLs suggested environmental effects on flowering responses. In silico analysis identified three candidate genes (*VsFTc*, *VsFTa*, and *VsLFY*) within flanking markers for stable QTL. This is the first report of QTLs for flowering in common vetch and provides targets for marker-assisted breeding to improve adaptation and resilience.

## Simple Summary

Common vetch (*Vicia sativa* L.) is one of the most important seed legumes widely used as a dual-purpose crop for forage, grain and green manure in cereal-based systems. Flowering time is a key trait because it determines crop adaptation, yield potential, and forage quality. In Mediterranean environments, early flowering is particularly important to avoid terminal drought and heat stress. However, despite its agronomic relevance, the genetic basis of flowering time in common vetch remains poorly understood. In this study, we evaluated a segregating population derived from two contrasting parental accessions during two consecutive growing seasons under Mediterranean field conditions. Flowering-related traits were assessed using thermal time, expressed as growing degree days, and quantitative trait loci (QTL) analysis was performed. To our knowledge, this is the first study reporting QTLs associated with flowering traits in common vetch. Nine QTLs were identified, five of which were consistently detected across both seasons, indicating their stability under contrasting environmental conditions. The potential role of candidate genes for flowering was also investigated using an in silico analysis. The genomic regions identified constitute valuable targets for marker-assisted breeding aimed at developing common vetch cultivars better adapted to Mediterranean environments and future climate change scenarios.

## 1. Introduction

Common vetch (*Vicia sativa* L.) is one of the most important annual seed legumes [1]. This self-pollinating species is widely used as a dual-purpose crop for forage, grain, and green manure in cereal-based cropping systems [1,2]. It provides high-protein biomass for grazing, hay or silage while improving soil fertility through biological nitrogen fixation and enhancing soil structure. It also contributes to weed and disease management when used in rotation or as a cover crop. Due to its ability to perform well under low-input conditions and on marginal soils, common vetch is increasingly recognized as a valuable component of sustainable and climate-resilient farming systems [3].

In 2024, the global production area dedicated to common vetch was 339,463 hectares, with a total production of 654,724 tonnes and an average yield of 1928.7 kg/ha [4]. Ethiopia was the world leader in both production and cultivated area. The crop is considered an excellent source of protein and minerals for animal feeding. It is less costly than other alternatives and has a high energy content and digestibility [1]. It can replace soybeans entirely or partially along with a large proportion of cereals in the diet [1]. Moreover, its suitability for intercropping systems [5] and its adaptation to diverse climate conditions and soils [1] make common vetch particularly attractive for sustainable agriculture in Mediterranean regions and suitable for the sustainable practices aimed for the European Union in both the Common Agricultural Policy and strategies such as From Farm to Fork.

Flowering time is one of the main determinants of harvesting time, which, in turn, is important for yield and the chemical composition in common vetch [6]. Crops have evolved sophisticated mechanisms to adjust flowering in response to environmental stimuli, mainly temperature and photoperiod, thereby maximizing reproductive success under different environmental conditions.

The importance of flowering time is particularly evident in Mediterranean environments, where terminal drought and heat stress frequently occur during the reproductive phase. Climate change scenarios predict that global warming levels will reach 1.4–4.4 °C by the end of the 21st century, alongside more frequent droughts and heat waves [7]. Under these conditions, early flowering genotypes are generally preferred because they can escape late-season stresses and maintain yield stability. Consequently, flowering time has become a major breeding target in Mediterranean cropping systems. In the case of common vetch, understanding the flowering process is considered crucial for breeding success [2]. The adaptive significance of this trait in common vetch has already been highlighted in studies comparing early- and late-flowering accessions, including transcriptomic analyses aimed at understanding the molecular basis of flowering regulation [7].

Despite its clear agronomic importance, flowering time in common vetch remains a poorly understood quantitative trait. Recent advances have focused on the genetic basis of traits such as drought and yield-related traits [8,9], salt tolerance [10], and biomass [11]. However, little is known about the genetic architecture underlying flowering. An exception is the study by Zhou et al. [12], which identified over four thousand differentially expressed genes between two common vetch accessions that differ in their flowering date.

This situation contrasts with that of other cool-season legumes. For example, studies on faba beans [13], lentils [14] and chickpeas [15,16] have demonstrated that dissecting the genetic architecture of flowering time and identifying stable loci across environments can significantly accelerate breeding for better adaptation and yield stability.

Despite the increasing availability of genomic and transcriptomic resources in common vetch, studies addressing the genetic control of flowering remain extremely limited. To our knowledge, no quantitative trait loci (QTL) analyses for flowering-related traits have been reported in this species. The recent availability of chromosome-level genome assemblies [17] and high-throughput genotyping platforms now provides unprecedented opportunities to dissect the genetic basis of flowering time and to develop molecular tools for breeding.

The objective of this study was to elucidate the architecture of flowering-related traits in common vetch under Mediterranean field conditions. To this end, QTLs controlling flowering were identified in an F_2:3_ population derived from two contrasting parental accessions and evaluated during two consecutive growing seasons. Specifically, we aimed to (*i*) identify QTLs associated with flowering-related traits, and (*ii*) validate these QTL by assessing their stability across seasons.

## 2. Materials and Methods

### 2.1. Plant Material, Data Collection and Field Trials

Two common vetch accessions, BG-23 and BG-24, were selected from the legume germplasm collection maintained at IFAPA-Centro Alameda del Obispo (Córdoba, Spain). These accessions were chosen because of their contrasting flowering behavior, with BG-23 exhibiting late flowering and BG-24 early flowering under Mediterranean field conditions.

An F_2_ population was developed from the cross BG-23/BG-24. A total of 87 F_2:3_ families, derived by selfing individual F_2_ plants, were evaluated under field conditions during two consecutive growing seasons (2023/24 and 2024/25) at IFAPA-Centro Alameda del Obispo, Córdoba, Spain (37°51′ N, 4°48′ W).

Field experiments were established using a randomized block design with three replications. In each replication, ten seeds per F_2:3_ family were sown in 1 m long rows with 50 cm spacing between rows.

Flowering-related traits were assessed using thermal time, expressed as growing degree days (GDD). Four traits were evaluated: (i) growing degree days to first flowering (GDDF), defined as the thermal time from sowing to the first flowering event within a row; (ii) growing degree days to complete flowering (GDDF100), corresponding to the thermal time from sowing until all plants within a row had flowered; (iii) flowering onset duration (GDDOF), defined as the thermal time elapsed between the appearance of the first flower in the earliest plant and the first flower in the last plant within a row; and (iv) flowering duration (GDDFD), defined as the thermal time from the first flowering event to the last flowering event recorded within a row.

### 2.2. Genetic Map Construction

Genomic DNA was isolated from young leaves following the CTAB protocol with slight modifications [18] using TissueLyser II mill (Qiagen, Hilden, Germany), two stainless-steel balls (5 mm diameter) for sample disruption and 2 mL Eppendorf tubes. Eighty-seven individuals from the BG-23/BG-24 population were used for map construction. Genotyping by sequencing analysis of the mapping population was performed by means of DArTSeq platform and aligned to the available chromosome-level assembly of the common vetch [17] by BLASTn (E-value 5 × 10^−7^, minimum sequence identify of 70% by Diversity Arrays Technology Pty Ltd. (Canberra, Australia). For mapping, only markers with a call rate above 90% and missing data below 7.5% were considered.

The genetic map was constructed using Joinmap^®^ v. 5.0 (Kyazma^®^, The Netherlands) using eighty-seven F_2_ individuals. Only DArTSeq markers with a call rate of above 90% and with consistent segregation in the parental lines of the population were used for mapping. Markers deviating from the ratio expected for mendelian segregation in an F_2_ population were excluded. Minimum LOD of 4.0 along with BLASTn results were used to assign markers to chromosomes. For each chromosome, several rounds of mapping were performed by excluding markers co-segregating in the same positions and using the EML algorithm (fastest). A final round of mapping was performed using the Regression Mapping algorithm (only second round of mapping) and the Kosambi mapping function to get the final maps.

### 2.3. Statistical and QTL Analysis

All statistical analyses were performed in RStudio v2024.04.1 Build 748. To evaluate Genotype × Environment (G × E) interactions, each flowering trait was analyzed across years using linear mixed-effect models (LMMs) implemented in lme4 package. Year, genotype (F_2:3_ family), and the Year × Genotype interaction were treated as fixed effects, whereas replication nested within year was considered as a random effect.

For each growing season, Best Linear Unbiased Estimators (BLUEs) for genotypic performance were estimated using the emmeans package, considering genotype as a fixed effect. Broad-sense heritability (H^2^) was estimated separately for each year from a fully random LMM using the equation: H^2^ = σ^2^g/(σ^2^g + σ^2^e/r), where σ^2^g is the genotypic variance, σ^2^e is the residual variance, and r corresponds to the number of replications (r = 3). Inter-annual temporal stability of flowering traits was assessed by calculating both Pearson’s correlation coefficient (r) and Spearman’s rank correlation coefficient (ρ) using genotype BLUEs from the two growing seasons.

QTL analyses were performed separately for each growing season using the BLUEs values for each trait and MapQTL^®^ v. 6.0 (Kyazma^®^, Wageningen, The Netherlands). In a first stage, marker–trait association were tested using the nonparametric Kruskal–Wallis test. After this, interval-mapping analyses were performed [19,20]. Finally, rMQM mapping was used [21,22,23]. The QTL significance (*p*-value) was calculated using a permutation test analysis (1000 permutations) [24]. QTL figures were generated by using MapChart software v2.32 [25]. The use of *p* values was reported as continuous quantities following the recommendations in [26]. Uncertainty in the QTL position was estimated using 1-LOD and 2-LOD support interval [20].

### 2.4. In Silico Identification and Selection of Flowering-Time Candidate Genes

Based on the differential response and regulatory patterns previously identified between contrasting *V. sativa* L. genotypes by Zhou et al. [12], a target panel of key flowering-time candidate genes was selected for genomic characterization. This selection comprised the *V. sativa* orthologs for the following candidate genes: *FLOWERING LOCUS T* (*VsFT*), *GIGANTEA* (*VsGI*), *PSEUDO*-*RESPONSE REGULATOR 5* (*VsPRR5*), *TIMING OF CAB EXPRESSION 1* (*VsTOC1*), *LEAFY* (*VsLFY*), and *SHORT VEGETATIVE PHASE* (*VsSVP*).

To retrieve robust query sequences for the in silico screening, homologous nucleotide and amino acid sequences were mined from phylogenetically closely related legume species, preferentially prioritizing characterized loci from *Pisum sativum* and *Medicago truncatula*. The complete list of reference species, source accessions, and sequence lengths utilized as queries is detailed in Table S1 (Supplementary Materials).

### 2.5. Homology Alignment, Physical Locus Annotation, and Marker Positioning

The retrieved legume query sequences were subjected to local alignment analyses using the Basic Local Alignment Search Tool (BLASTn and, BLAST+ 2.17.0) against the official *V. sativa* reference genome assembly, hosted at the National Center for Biotechnology Information (NCBI) under the BioProject accession PRJNA762450 (isolate South Australia). To ensure a highly rigorous identification of true orthologs, high-scoring segment pairs (HSPs) were filtered using stringent thresholds, requiring an E-value ≤ 10^−20^ and high query coverage. The absolute physical boundaries of each successfully anchored locus—including chromosome assignment, start/end base pair (bp) coordinates, and strand orientation—were obtained. Concurrently, the absolute physical coordinates of the molecular markers constituting the linkage map were provided by Diversity Arrays Technology Pty Ltd. (DArT, Canberra, Australia), which were determined by aligning the obtained trimmed sequences against the same *V. sativa* reference genome assembly as explained above.

## 3. Results

### 3.1. Construction of the Genetic Map

An F_2_ population derived from the cross BG-23/BG-24 consisting of eighty-seven individuals was obtained and used for map construction. The mapping population was genotyped using the DArTSeq platform and the sequences aligned to the common vetch reference genome (Diversity Array Technology Pty Ltd., DArT P/L, Canberra, Australia). After filtering out markers with a call rate below 90% and missing data exceeding 7.5%, an initial dataset of 10,891 markers, including 7022 silico DArT and 3869 SNPs, was considered for map construction. The final map contained 419 unique positions (bin markers) distributed in six linkage groups corresponding to *V. sativa* chromosomes.

The genetic map spanned a total distance of 1728.5 cM, with an average inter-marker distance of 3.68 cM. The inter-marker distance varied between 2.85 for chromosome 2 and 5.21 for chromosome 3, with the largest gap in the map (49.1 cM) found in chromosome 5.

### 3.2. Climatic Conditions

**Figure 1:**
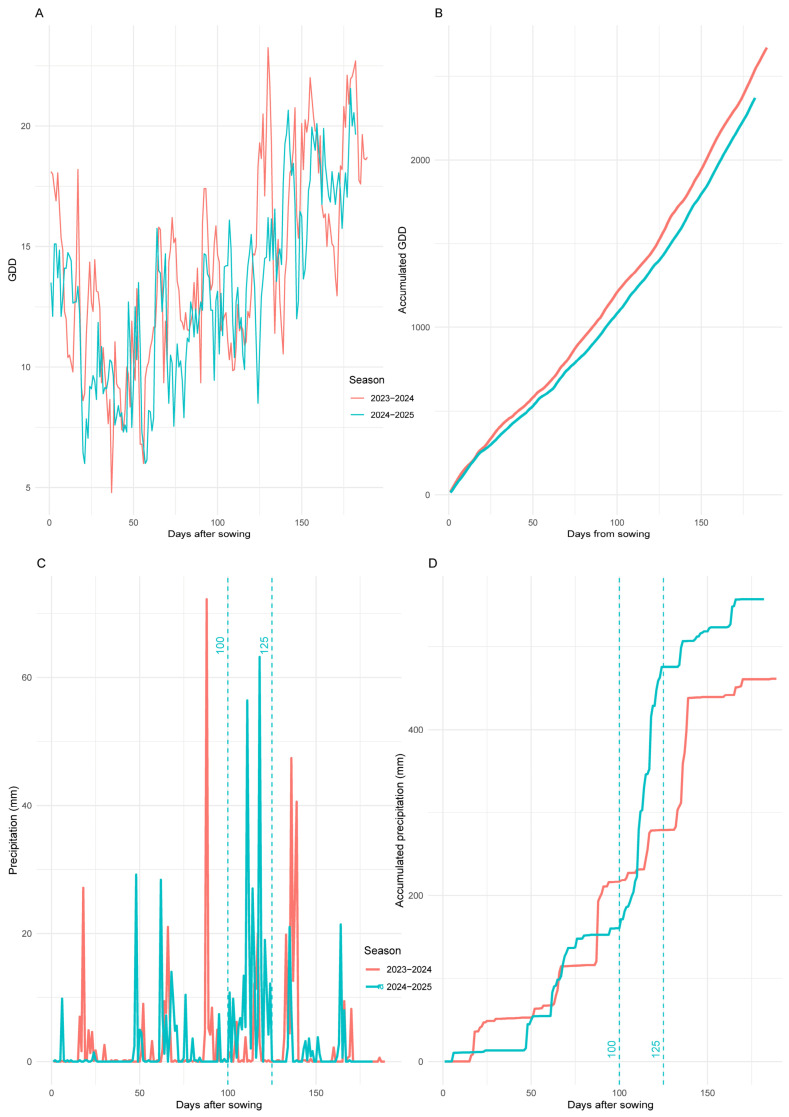
Overview of temperature and precipitation dynamics. (**A**) Daily growing degree days. (**B**) Accumulated growing degree days. (**C**) Daily precipitation. (**D**) Accumulated precipitation. The vertical lines in the precipitation figures denote the heavy rain episode during the 2024/25 season.

The two growing seasons differed markedly in their climatic conditions (Figure 1). The 2023/24 season was generally warmer and exhibited greater day-to-day temperature fluctuations, resulting in higher cumulative growing degree days throughout the crop cycle (Figure 1A,B). In contrast, the 2024/25 season was characterized by lower thermal accumulation and a more uniform temperature pattern.

Precipitation patterns also differed substantially between seasons (Figure 1C,D). While rainfall during the 2023/24 season was relatively evenly distributed, the 2024/25 season experienced an intense rainfall episode shortly before flowering, resulting in the accumulation of 314.8 mm of precipitation between 100 and 125 days after sowing. This rainfall event temporarily altered field conditions and delayed crop development, leading to a later onset of flowering during the second season (Figure 1C,D).

Overall, the contrasting temperature and precipitation regimes observed between seasons provided a suitable environmental framework for assessing the stability of flowering-related traits and associated QTLs under Mediterranean field conditions.

### 3.3. Phenotypic Assessment and Descriptive Statistics

The BG-23/BG-24 F_2:3_ population was evaluated for flowering-related traits under field conditions during the 2023/24 and 2024/25 growing seasons. Flowering phenology was assessed using thermal time, expressed as growing degree days (GDD), which is a measure of developmental progress based on the amount of heat accumulated during the crop cycle. The following four traits were evaluated: initial flowering (GDDF); final flowering (GDDF100); time between first flowering event in the earliest and the latest plant within a row (GDDOF); and total flowering duration, defined as the period from the first flower in the first plant to the last flowering event within the row (GDDFD). Descriptive statistics for these traits can be found in Table 1.

Substantial phenotypic variation was observed for all traits in both growing seasons, indicating the suitability of the BG-23/BG-24 population for genetic analysis. The highest broad-sense heritability estimates were obtained for GDDF and GDDF100, with H^2^ values ranging from 0.79 to 0.86 and from 0.70 to 0.80, respectively, suggesting a strong genetic control of flowering initiation and completion. In contrast, lower heritability estimates were observed for GDDOF and GDDFD, particularly during the 2024/25 season, indicating a stronger environmental influence on flowering duration traits.

**Figure 2:**
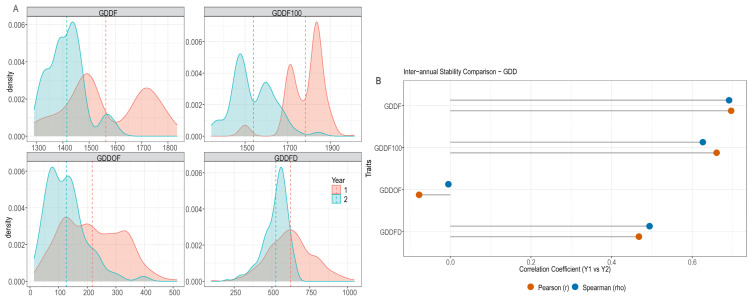
Phenotypic assessment of the BG-23/BG-24 population over two seasons. (**A**) Density functions showing the distribution of traits during both seasons. Dashed lines show mean values in each season. (**B**) Inter-annual correlations (Pearson’s and Spearman’s) for flowering traits. Year 1 corresponds to the 2023/24 season, and Year 2 corresponds to the 2024/25 season.

Frequency distributions for all traits showed continuous variation supporting the quantitative nature of flowering phenology in common vetch (Figure 2A). Trait distributions were generally comparable across seasons, although flowering occurred later during the 2024/25 season, consistent with the contrasting climatic conditions observed between years.

Inter-annual correlations revealed high temporal stability for GDDF and GDDF100, with both Pearson’s and Spearman’s correlation coefficients exceeding 0.60 (Figure 2B). Moderate correlations were observed for GDDFD, whereas GDDOF showed no significant correlation between seasons. These results indicate that the onset and completion of flowering are relatively stable traits across environments, while flowering duration appears to be more sensitive to environmental variation.

Overall, high inter-annual Pearson’s and Spearman’s rank correlations were found for GDDF and GDDF100 with values above 0.6. Moderate values below 0.5 were obtained for GGDFD. Finally, no correlation was found for GGDOF (Figure 2).

### 3.4. QTL Detection

A total of ten QTLs associated with flowering-related traits were detected on chromosomes 2, 3, 4 and 6 (Table 2). The proportion of phenotypic variance explained (R^2^) by individual QTLs ranged from 9.1% to 40.4%, indicating the presence of both major- and moderate-effect loci controlling flowering phenology in common vetch.

**Figure 3:**
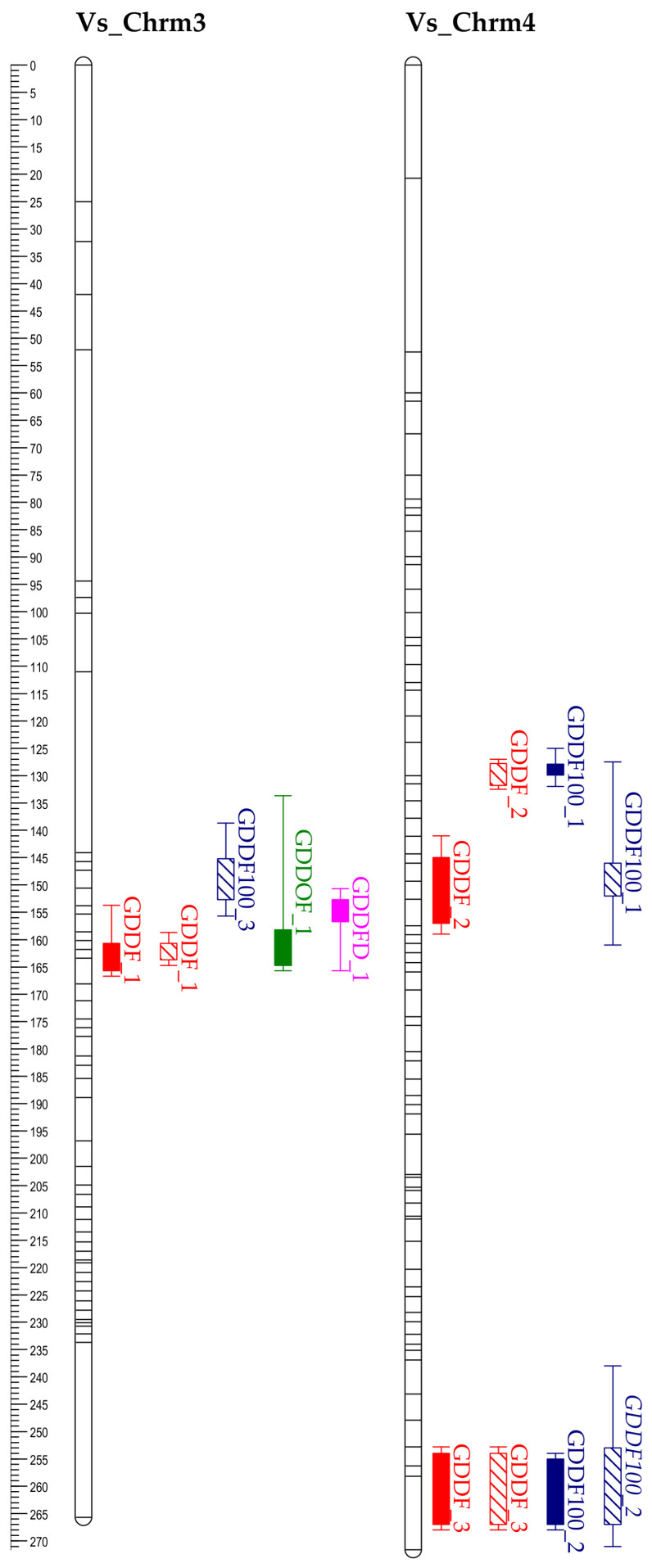
Location of the three genomic validated regions for flowering traits in the BG-23/BG-24 population (genetic distance in cM is shown on the left). QTLs are shown as bars for the 1-LOD interval and as whiskers for the 2-LOD interval. The QTLs corresponding to the 2023/24 season are shown with a solid fill, and those corresponding to the 2024/25 season are shown with a hatched fill.

Five QTLs, namely GDDF_1, GDDF_2, GDDF_3, GDDF100_1 and GDDF100_2 were consistently detected across both growing seasons. These stable QTLs were located in three major genomic regions located on chromosomes 3 and 4 (Figure 3), suggesting that these regions represent key determinants of flowering phenology in the BG-23/BG-24 populations (Figure 3).

Additional QTLs were detected in only one growing season. Nevertheless, GDDF100_3, GDDOF_1 and GDDFD_1 co-localized with stable QTLs identified on chromosome 3, reinforcing the biological relevance of this genomic regions. In contrast, GDDOF_2 and GDDOF_3, identified on chromosomes 2 and 6, respectively, were detected exclusively during the 2024/25 season and were therefore considered putative QTLs requiring further validation.

### 3.5. Genomic Identification of Candidate Genes and Spatial Colocalization with Flowering QTLs

A set of candidate genes (see Table S1) was selected because they have been reported to be potentially involved in the process of regulating flowering in *V. sativa*, as revealed by transcriptomic and metabolomic studies [12].

Flowering-time candidate genes (Table S1) were aligned to the *V. sativa* reference genome. The BLAST (BLAST+ 2.17.0) results for all the candidate genes, including the physical positions in common vetch, are shown in Table S2. The sole exception to this screening was the PSEUDO-RESPONSE REGULATOR 5 (PRR5) gene; despite retrieving and evaluating its orthologous sequence from *Arabidopsis thaliana*, it failed to yield significant alignments against standard reference legume sequences and did not show any valid homology or alignment blocks against the *V. sativa* reference genome, thereby preventing its physical annotation.

As explained in Section 2, DArTSeq markers were successfully aligned against *V. sativa* genome. The physical position of flanking markers of QTLs identified in this work was considered to investigate whether these intervals included any of the candidate genes evaluated. This analysis revealed that *FLOWERING LOCUS T* a (*VsFTa*) and *FLOWERING LOCUS T c* (*VsFTc*) were within the confidence interval of the overlapping QTLs GDDF_2 and GDDF100_1 on chromosome 4. Similarly, the *LEAFY* (*VsLFY*) gene was also positioned within the interval defined by the flanking markers of QTLs GDDF100_3 and GDDOF_1 on chromosome 3. Table S3 indicates the physical position of these genes along with the flanking markers for these QTLs and Figure S1 shows their estimated position in the BG-23/BG-24 genetic map.

## 4. Discussion

Flowering time is a key adaptive trait in crop species because it determines the transition from vegetative phase to reproductive period and strongly influences crop adaptation, yield potential, and forage quality. The accumulation of thermal time above a base temperature is known to regulate phenological development in many crops, including common vetch [27]. However, many agronomy, breeding, and QTL studies still rely on measuring flowering in calendar days from sowing because of the simplicity and widespread use of this approach. Indeed, many QTL studies in legumes are based on flowering time measured in days, as is the case in faba bean [13,28,29], lentil [14] or chickpea [16]. Although QTL positions are expected to be largely conserved regardless of the phenological scale used, the use of thermal time provides a more physiologically meaningful measure of plant development and facilitates comparisons among environments differing in temperature regimes. Therefore, assessing flowering-related traits as growing degree days (GDD) may improve the identification of genomic regions associated with phenological adaptation.

In the present study, three genomic regions were consistently detected over two seasons: one in chromosome 3 and two in chromosome 4, indicating that these regions constitute the main determinants of flowering in the BG-23/BG-24 population. The identification of stable QTLs across different climatic conditions is particularly relevant for breeding programs because such loci are more likely to confer broad adaptation under variable climatic conditions [30]. Despite the marked differences in temperature and precipitation observed between seasons, these genomic regions remained consistently associated with flowering traits, suggesting that their effects are largely independent of environmental fluctuations. The stability of these QTLs highlights their potential usefulness for marker-assisted selection aimed at improving adaptation in Mediterranean environments.

Flowering time has been extensively studied in several legume species, particularly pea and soybean, but also in cool-season legumes such as faba bean, lentil, and chickpea [13,31,32]. In these species, flowering phenology is typically controlled by a limited number of major loci together with additional loci of smaller effect. The identification of three major genomic regions in common vetch is therefore consistent with the genetic architecture reported in other legumes. For example, up to eight regions of the faba bean genome spanning six linkage groups were identified in the Icarus/Ascot population in relation to flowering time, in terms of both days to flowering and thermal units [31]. Similarly, a total of six QTLs for flowering time distributed over four chromosomes were also identified in faba bean [13]. The results obtained in the present study suggest that flowering phenology in common vetch is also governed by a relatively simple genetic architecture dominated by a few major-effect loci. To our knowledge, this is the first study reporting QTLs associated with flowering-related traits in common vetch, thus providing a first framework for the genetic dissection of this trait in the species.

The onset of flowering and the duration of flowering are not equivalent traits. The indeterminate growth habit of common vetch implies simultaneous vegetative and reproductive growth once flowering has started. Consequently, the date of flowering does not provide information on how long reproduction takes place. Indeed, flowering initiation and flowering duration have been shown to be physiologically distinct traits with partially independent genetic controls in other legumes, such as mungbean [33] and soybean [34]. In our study, QTLs on chromosome 4 were exclusively associated with flowering onset traits, whereas the genomic region identified on chromosome 3 harbored QTLs controlling both flowering time and flowering duration. These results suggest the existence of at least partially independent genetic systems regulating these traits in common vetch, as previously described in other legumes [33,34].

The two growing seasons differed substantially in their climatic conditions, particularly in terms of temperature and precipitation patterns. The second season was characterized by an intense rainfall episode before flowering, which likely caused temporary soil waterlogging and delayed crop development. Waterlogging is known to impair growth and physiological processes in legumes, and common vetch has been reported as one of the forage legume species most sensitive to flooding conditions [35]. Flooding events have also been shown to delay phenological development in soybean [36]. This is particularly important in common vetch, as it is the species most sensitive to waterlogging among forage legumes [35]. Consequently, the season-specific QTLs identified for flowering duration traits may reflect genotype-specific responses to environmental conditions rather than constitutive flowering mechanisms. These QTLs should therefore be considered putative and require further validation under additional environments and controlled stress conditions.

The physical colocalization of *VsFTa*, *VsFTc*, and *VsLFY* within the confidence intervals of QTLs on chromosomes 3 and 4 strongly highlights these loci as primary positional and functional candidate genes driving the observed variations in flowering time in these chromosome regions. The identification of *VsFTa* and *VsFTc* within the overlapping intervals of QTLs GDDF_2 and GDDF100_1 on chromosome 4 represents a highly consistent finding within legume comparative genomics.

Given their well-documented roles in integrating environmental signals for flowering and in signaling from the site of photoperiod detection in the leaf to the site of flower formation at the shot apex, FT genes are of particular interest for understanding the control of flowering time in legumes [32]. Functional characterization in model legumes has revealed that the FT family underwent an ancient duplication event, evolving into distinct subclades (*FTa*, *FTb*, and *FTc*) with specialized photoperiodic and regulatory roles [37]. The tight physical clustering of *VsFTa* and *VsFTc* on chromosome 4 mirrors the genomic architecture observed in other Papilionoideae species, suggesting that this locus represents a highly conserved genomic hotspot controlling the vegetative-to-reproductive transition in *V. sativa* [37].

Equally significant is the positioning of *VsLFY* within the interval defined by the flanking markers of QTLs GDDF100_3 and GDDOF_1 on chromosome 3. The *LEAFY* (*LFY*) gene functions in floral meristem identity control during reproductive phase [38]. In pea, the functional ortholog of *LFY* is the *UNIFOLIATA* (*UNI*) gene. It regulates flower morphogesis and it plays crucial roles in both specifying floral meristem identity and governing compound leaf morphogenesis [39]. Mutations or regulatory alterations at these loci drastically disrupt floral initiation and timing [38,39]. Consequently, the presence of a robust *VsLFY* ortholog within the chromosome 3 region harboring QTLs provides a solid biological foundation to explain the genetic variance associated with this locus.

While *VsFTa*, *VsFTc*, and *VsLFY* stand out as excellent candidate genes, further validation is required in future investigations. At present, A RIL population derived from the cross used in this study in under development and it will provide a valuable and stable genetic background required for high-resolution fine-mapping and definitive statistical validation of the candidate loci. Future research combining fine mapping, comparative genomics, and transcriptomics will contribute to elucidating the molecular mechanisms underlying flowering phenology in this species and accelerate the development of molecular tools for breeding.

As an immediate next step, it will be important to target the genomic region surrounding *VsLFY* on Chromosome 3. As demonstrated by our comparative physical-genetic framework, the *VsLFY* locus (positioned at 222.05 Mb) resides within the flanking markers of the QTLs but it is within a gap spanning 33 cM in the genetic map. Future work must focus on saturating this specific interval. Utilizing the reference assembly as a template, targeted locus-specific polymorphic markers—such as simple sequence repeats (SSRs) or insertions/deletions (InDels)—should be developed to populate this gap. Saturating this region within the upcoming RIL population will compress the QTL confidence interval, resolve local recombination rates, and conclusively establish whether sequence or expression polymorphisms at the *VsLFY* locus are the direct causative drivers of the target phenological trait in *V. sativa*.

## 5. Conclusions

This study provides the first insight into the genetic architecture of flowering-related traits in common vetch through QTL analysis under Mediterranean field conditions. A high-density genetic map enabled the identification of ten QTLs associated with flowering phenology, of which five were consistently detected across two contrasting growing seasons. These stable QTLs defined three major genomic regions that appear to play a central role in the control of flowering in the BG-23/BG-24 population.

The identification of genomic regions consistently expressed across environments is particularly relevant for breeding programs aimed at broad adaptation under Mediterranean conditions. In addition, season-specific QTLs suggest that environmental factors, particularly those related to water availability, may influence flowering dynamics through specific genomic regions.

The genomic regions identified in this study constitute valuable targets for future fine mapping, candidate gene identification, and marker-assisted breeding. Overall, these findings contribute to a better understanding of flowering regulation in common vetch and provide useful genomic tools for the development of cultivars better adapted to future climate scenarios.

## Figures and Tables

**Table 1:** Phenotypic assessment of the BG-23/BG-24 population.

Trait ^1^	Year	Mean ^2^	Range	CV(%) ^3^	Broad Sense Heritability (H^2^)
GDDF	2023	1563.4 ± 148.7	1290–1835	9.5	0.86
GDDF	2024	1414.9 ± 70.8	1300–1625	5	0.79
GDDF100	2023	1782.7 ± 88.6	1499–2013	5	0.8
GDDF100	2024	1538.5 ± 99.8	1341–1843	6.5	0.7
GDDOF	2023	217.4 ± 103	36–514	47.4	0.58
GDDOF	2024	126.8 ± 70.1	14–401	55.3	0.26
GDDFD	2023	623.1 ± 149.2	99–1045	23.9	0.38
GDDFD	2024	525.4 ± 80.5	205–661	15.3	0.59

^1^ Traits. GDDF (growing degree days to initial flowering); GDDF100 (growing degree days to 100% of flowering); GDDOF (growing degree days of flowering, from 1st flower in the 1st plant, to the 1st flower in the last plant in the row); GDDFD (growing degree days of flowering, from the 1st to the last flowering event in the row. ^2^ Mean ± standard deviation. ^3^ Coefficient of variation (%).

**Table 2:** QTL for flowering traits in the BG-23/BG-24 population.

Year	QTL_Name ^1^	Chm ^2^	LOD	Position (cM)	Flanking Markers	*p*-Value ^3^	R^2^ (%)
2023	GDDF_1	3	6.09	102.3	100059540/100059233	0.003	32.1
2023	GDDF_2	4	7.98	149.3	100153637/100126444	0.000	13.7
2023	GDDF_3	4	4.76	258	100156464/100150543	0.017	9.1
2024	GDDF_1	3	5.1	103.93	100145069/100059233	0.002	39.6
2024	GDDF_2	4	5.01	157.46	100199025/100231196	0.003	21.1
2024	GDDF_3	4	5.15	258	100156464/100150543	0.002	21.5
2023	GDDF100_1	4	9.95	128.92	100199025/100126518	0.000	40.3
2023	GDDF100_2	4	6.42	263.2	100064638/100150543	0.002	40.4
2024	GDDF100_3	3	4.12	118.4	100168293/100145069	0.014	20.4
2024	GDDF100_1	4	4.1	149.3	100199025/100166081	0.015	27.4
2024	GDDF100_2	4	2.85	258	100171611/100150543	0.174	10.5
2023	GDDOF_1	3	5.93	102.3	100168293/100059233	0.002	29.3
2024	GDDOF_2	2	4.39	73.1	100002293/100171609	0.009	17.5
2024	GDDOF_3	6	3.66	64.7	100130600/100144489	0.041	14.9
2023	GDDFD_1	3	6.18	110.4	100059540/100059233	0.000	23.4

^1^ Traits abbreviations: GDDF (growing degree days to initial flowering); GDDF100 (growing degree days to 100% of flowering); GDDOF (growing degree days of flowering, from 1st flower in the 1st plant, to the 1st flower in the last plant in the row); GDDFD (growing degree days of flowering, from the 1st to the last flowering event in the row. ^2^ Chm: Chromosome. ^3^ QTL probability determined from the permutation test.

## Data Availability

Data are contained within this article and the Supplementary Materials.

## Supplementary Materials

The following supporting information can be downloaded at: https://www.mdpi.com/article/10.3390/biology15141209/s1, Table S1: List of gene sequences used for BLASTn and BLASTx against the official *V. sativa* reference genome. Table S2: BLAST results for the candidate genes indicated in Table S1. Table S3: Position of candidate genes between the flanking markers of QTLs detected in this study. Figure S1: Estimated position of candidate genes in the BG-23/BG-24 genetic map.

## Abbreviations

The following abbreviations are used in this manuscript:

BLUEs	Best Linear Unbiased Estimators
GGDs	Growing degree days
GDDF	Growing degree days to first flowering
GDDF100	Growing degree days to complete flowering
GDDOF	Flowering onset duration
GDDFD	Flowering duration
*LFY*	Leafy
QTL	Quantitative trait locus
RIL	Recombinant inbred line
*VsFt*	*Vicia sativa FLOWERING LOCUS T*
