# Supplementary material for: Unravelling the Genetic Architecture of Flowering in Common Vetch (Vicia sativa L.) Through Multi-Season QTL Mapping
Source: Biology (Basel). 2026 Jul 22;15(14):1209. doi: 10.3390/biology15141209 (PMC13404190; doi:10.3390/biology15141209)

Table S1. List of gene sequences used for BLASTn and BLASTx against the official *V. sativa* reference genome.

Putative gene	Query Species &Accesions
VsFTa	*P. sativum* _ HQ538830.1
VsFTb1	*P. sativum* _ HQ538831.1
VsFTb2	*P. sativum* _ HQ538831.1
VsFTc	*P. sativum* _ HQ538832
VsGI	*M.truncatula* _ XM_024786082.2
VsTOC1_5A	*P. sativum* _ AY830927.1
VsTOC1_5B	*P. sativum* _ AY830927.1
VsTOC1_3	*P. sativum* _ AY830927.1
VsLFY	*M.truncatula* _ JF681134.1
VsSVP	*C.arietinum* _ XM_012718387
VsPRR5	*A. thaliana _ NM_122355.4*

Table S2. BLAST results for the candidate genes indicated in Table S1

Putative gene	Query Species &Accesions	Subject ID (NCBI)	Chr Vs	Start (bp)	End (bp)	Strand	E-value	Identity (%)	Query Cover (%)
VsFTa	*P. sativum* _ HQ538830.1	CM038795.1	4	156,281,546	156,282,706	Minus	5E-160	86.05%	86%
VsFTb1	*P. sativum* _ HQ538831.1	CM038793.1	2	3,087,473	3,089,980	Minus	9E-117	87.17%	30%
VsFTb2	*P. sativum* _ HQ538831.1	CM038793.1	2	3,152,051	3,154,977	Plus	4E-105	88.00%	30%
VsFTc	*P. sativum* _ HQ538832	CM038795.1	4	156,070,076	156,078,573	Minus	0.0	85.35%	46%
VsGI	*M.truncatula* _ XM_024786082.2	CM038792.1	1	88,911,156	88,918,551	Minus	0.0	93.98%	90%
VsTOC1_5A	*P. sativum* _ AY830927.1	CM038796.1	5	214,406,848	214,407,008	Plus	3E−59	92.55%	100%
VsTOC1_5B	*P. sativum* _ AY830927.1	CM038796.1	5	214,414,034	214,414,184	Plus	3E−55	92.71%	100%
VsTOC1_3	*P. sativum* _ AY830927.1	CM038794.1	3	24,222,204	24,222,364	Minus	3E−54	90.68%	100%
VsLFY	*M.truncatula* _ JF681134.1	CM038794.1	3	222,057,987	222,059,914	Minus	1E−155	88.62%	100%
VsSVP	*C.arietinum* _ XM_012718387	CM038793.1	2	249,655,304	249,656,870	Minus	7E−54	89.94%	35%
VsPRR5	*A. thaliana _ NM_122355.4*	-	-	-	-	-	-	-	-

Table S3. Position of candidate genes between the flanking markers of QTLs detected in this study.

Candidate gene	Start Position (bp)	End Position	Vs Chromosome	QTL	Flanking marker up	Marker sequence	cM map position	Physical position (bp	Flanking marker down	Marker sequence	cM map posi	ChromPosTag_Vicia_sati
VsFTc	156,070,076	156,078,573	4	GDDF_2, GDDF100_1	100153637	TGCAGCAGGTTAA	137.8	134,583,773	100000858	TGCAGTAGTATTA	141.1	163,796,176
VsFTa	156,281,546	156,282,706	4	GDDF_2, GDDF100_1	100153637	TGCAGCAGGTTAA	137.8	134,583,773	100000858	TGCAGTAGTATTA	141.1	163,796,176
VsLFY	222,057,987	222,059,914	3	GDDF100_3, GDDOF_1	100168293	TGCAGCCTCTCAA	111.0	176,690,340	100153657	TGCAGCAGTGCA	144.1	258,200,166

Figure S1. Estimated position of candidate genes in the BG-23/BG-24 genetic map.

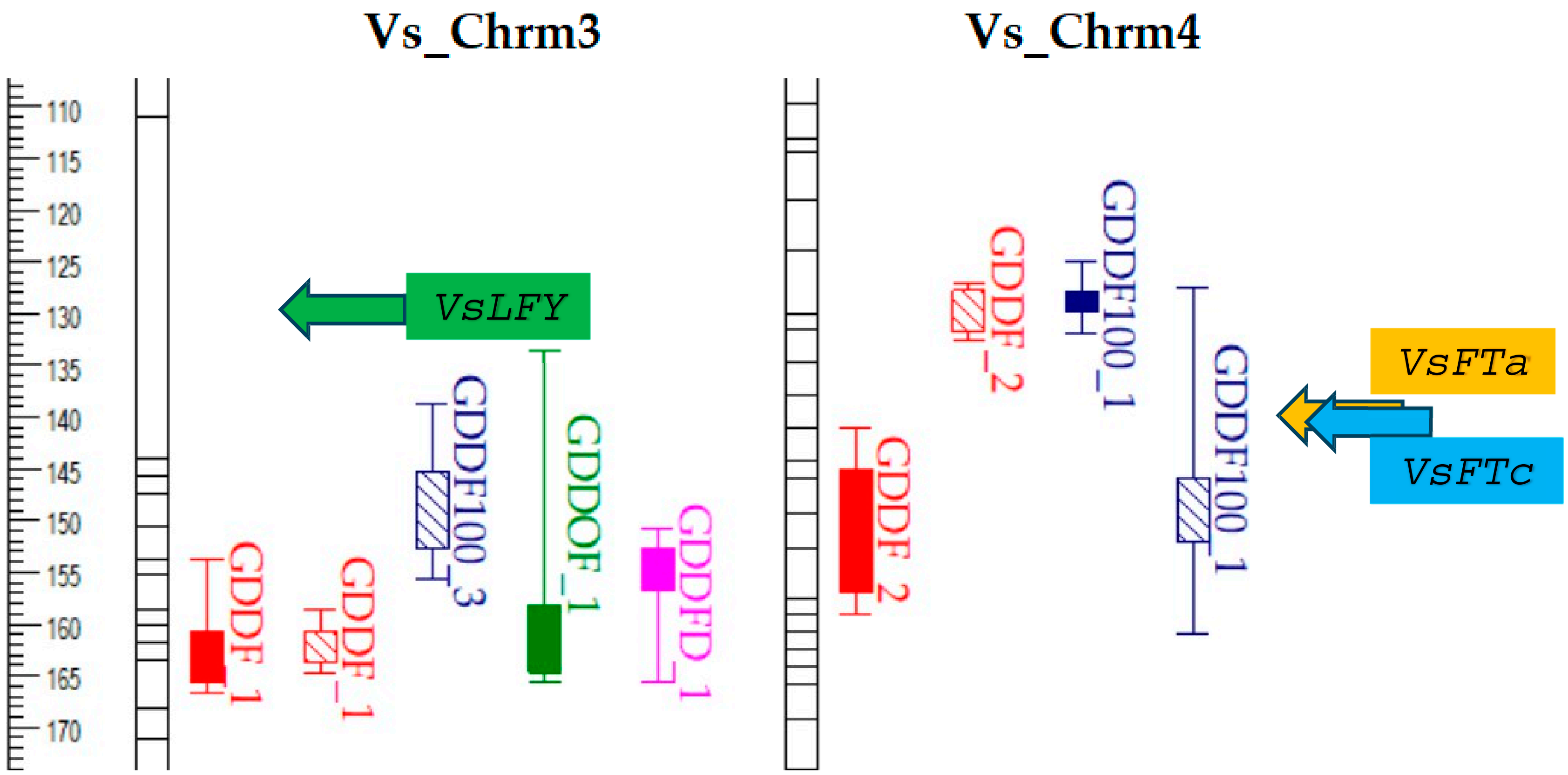

Supplement: Supplementary file 1 [file biology-15-01209-s001.zip › biology-4436057-supplementary.pdf]
